# Overcoming resistance barriers: Using the salvage therapy of Ceftazidime-Avibactam and Aztreonam for difficult-to-treat gram-negative infections at a tertiary care hospital in Karachi-A retrospective cohort pilot

**DOI:** 10.12669/pjms.42.(ICON26).15693

**Published:** 2026-04

**Authors:** Samreen Sarfaraz, Syeda Mahnoor Zafar, Nabeel Ahmed Siddiqui, Muhammad Hamid Hanif, Zahid Hussain

**Affiliations:** 1 Samreen Sarfaraz, FRCP. Chair of Department of Infectious Diseases, Indus Hospital & Health Network, Korangi, Karachi, Pakistan; 2 Syeda Mahnoor Zafar, Pharm-D. Senior Research Scientist, ORIC, Indus Hospital & Health Network, Korangi, Karachi, Pakistan; 3 Nabeel Ahmed Siddiqui, Pharm-D. Research Associate, ORIC, Indus Hospital & Health Network, Korangi, Karachi, Pakistan; 4 Muhammad Hamid Hanif, Pharm-D. Clinical Pharmacist, Department of Infectious Disease, Indus Hospital & Health Network, Korangi, Karachi, Pakistan; 5 Dr. Zahid Hussain, FCPS. Resident, Department of Infectious Disease, Indus Hospital & Health Network, Korangi, Karachi, Pakistan

**Keywords:** Aztreonam_5_, Carbapenem-Resistant Enterobacteriaceae (CRE)_1_, Ceftazidime-Avibactam_4_, Combinational therapy_2_, Pakistan, Salvage therapy_3_

## Abstract

**Background & Objective:**

Rise of carbapenem resistance poses a major global health challenge, particularly in Pakistan where treatment options for Gram-negative carbapenem-resistant Enterobacteriaceae (CRE), are extremely limited. Last-resort agents such as colistin, tigecycline, and fosfomycin are constrained by toxicity and emerging resistance. Aztreonam, active against metallo-β-lactamase–producing organisms, is often unavailable or unaffordable. At Indus Hospital and Health Network (IHHN), Ceftazidime-Avibactam (CAZ-AVI) combined with Aztreonam (ATM) was used as salvage therapy between September 2023 and September 2024. The study aimed to evaluate outcomes of using CAZ-AVI with Aztreonam in patients with CRE.

**Methodology:**

This retrospective cohort study was conducted at IHHN from September 2023 to September 2024. Patients with carbapenem-resistant infections treated with CAZ-AVI + ATM were included, excluding CRAB. Due to limited availability of ATM in Pakistan, only 21 patients received this regimen, enabling it as pilot study. Clinical and microbiological data were extracted from HMIS and analyzed using SPSS.

**Results:**

Among 21 treated patients, the regimen yielded an in-hospital survival rate of 85.7% and clinical improvement in 83% of survivors, with minimal adverse effects. These findings suggest meaningful clinical benefit in patients with limited therapeutic options.

**Conclusion:**

CAZ-AVI plus ATM appears to be an effective salvage therapy for carbapenem-resistant Gram-negative infections in resource-limited settings. The study also underscores a need for reliable aztreonam availability in tertiary care hospitals. Significant findings yielded from this limited data set calls for larger prospective comparative studies to further validate these results.

## INTRODUCTION

Global spread of Carbapenem-resistant *Enterobacteriaceae* (CRE) has become a pressing global health concern, disproportionately affecting low-income countries such as Pakistan,^1^ and resulting in high morbidity and mortality.^2^ Reported CRE prevalence ranges from 21.8% in a tertiary care hospital in Karachi to 31.8% in regional studies from South Asia.^2^-^4^ Similarly, *Pseudomonas aeruginosa* has demonstrated increasing multidrug resistance over the past two decades, significantly compromising the effectiveness of carbapenems such as meropenem.^5^,^6^

Metallo-β-lactamase (MBL)–producing organisms, particularly those harboring New Delhi metallo-β-lactamase (NDM), pose a critical therapeutic challenge due to resistance to nearly all available antibiotics.^7^ Studies from Karachi report MBL production in up to 82.7% of *Enterobacteriaceae* isolates.^4^ Despite this high burden, carbapenemase detection remains limited to a few specialized laboratories in Pakistan, restricting optimal antimicrobial selection. The Infectious Diseases Society of America (IDSA) recommends molecular or antigen-based carbapenemase testing to guide therapy in these highly resistant infections.^8^

Ceftazidime-avibactam (CAZ-AVI) is recommended for infections caused by KPC- and OXA-48–producing organisms and has demonstrated improved outcomes and lower toxicity compared with colistin-based regimens.^8^,^9^ However, it lacks activity against MBL-producing pathogens.^10^

In contrast, combination of CAZ-AVI with aztreonam (ATM) exhibits synergistic activity by inhibiting a broad spectrum of β-lactamases, restoring antibacterial activity even in colistin-resistant isolates.^11^ This combination, along with cefiderocol, is recommended for treating MBL-producing CRE, though cefiderocol is currently unavailable in Pakistan.^8^

Due to interrupted access to aztreonam, clinicians in Pakistan are often forced to rely on less effective and more toxic alternatives such as colistin, tigecycline, and fosfomycin, despite rising resistance rates.^8^

The Infectious Disease Department at IHHN utilized Ceftazidime-Avibactam (CAZ-AVI) in combination with Aztreonam (ATM) as a last-resort therapy for patients with carbapenem-resistant Gram-negative infections who had no other viable antibiotic options due to extensive resistance or severe adverse reactions. Owing to recurring shortages of Aztreonam, only 21 patients were able to receive this life-saving combination therapy over the course of one year.

This retrospective pilot study evaluated clinical outcomes and underscores the potential role of this combination in managing otherwise untreatable infections, while highlighting an urgent need for reliable aztreonam availability and larger prospective studies.

## METHODOLOGY

It is a retrospective cohort pilot study with non-probability consecutive sampling. Patients with Carbapenem resistant Enterobacteriaceae infections visited or hospitalized at Indus Hospital & Health Network, Korangi.Patient data were collected from the duration of September 2023 to September 2024 from the Hospital’s Health Management Information System (HMIS) with help from a pre-designed questionnaire.

### Ethical Approval:

This study was carried out after approval from the IRB with registration no. IHHN_IRB_2024_10_002; dated: October 10, 2024.

### Inclusion criteria:

- All patients who were given Ceftazidime/Avibactam with Aztreonam as a salvage therapy against Carbapenem-resistant gram-negative infection were included.

### Exclusion criteria:

- All patients infected with carbapenem-resistant *Acinetobacter baumannii* (CRAB) were excluded.

***Details of drug:*** In this study, in case of normal renal function ceftazidime avibactam 2.5g Q8H IV and aztreonam 2g q8h IV was given to patients, both these drugs administered simultaneously over 3 hours. In case of acute kidney injury, creatinine clarence was calculated by using Cockcroft gault equation and renal adjusted dose was given as per creatinine clarence, following are renal dose adjustment which was followed:

### For Ceftazidime + avibactam:

- CrCl >30 to 50 mL/minute: 1.25 g every 8 hours.
- CrCl >15 to 30 mL/minute: 0.94 g every 12 hours.
- CrCl >5 to 15 mL/minute: 0.94 g every 24 hours.
- CrCl ≤5 mL/minute: 0.94 g every 48 hours

### For Aztreonem:

- CrCl 10 to <30 mL/minute: 2 g every 12 hours
- CrCl <10 mL/minute: 2 g every 24 hours

### Data collection:

A study-specific questionnaire was developed and used to extract data from the IHHN’s HMIS. Variables included demographics, infection characteristics, comorbidities, microbiological findings, and treatment details. Outcomes assessed were survival, clinical improvement based on symptom resolution and normalization of infection markers, culture clearance in cases of bacteremia, adverse events, and follow-up clinical status. This approach ensured standardized and comprehensive data collection for all 21 patients who received ceftazidime-avibactam plus aztreonam salvage therapy.

### Statistical analysis:

Data analysis was conducted using SPSS version 26. The Shapiro-Wilk test assessed normality of continuous variables, which are presented as mean ± standard deviation (SD) if normally distributed, or median with interquartile range (IQR) if non-normally distributed. Categorical variables are expressed as frequencies and percentages. Primary outcome was in-hospital survival, while secondary outcomes included clinical improvement at the end of treatment, 30-day relapse rate, and incidence of treatment-related adverse events. Clinical improvement was determined based on the treating physician’s assessment.

## RESULTS

### Clinical and Demographic Characteristics of Study Participants:

The 21 participants included were predominantly male (66.7%) with a mean age of 37 years and a median monthly income of PKR 60,000. Nearly half (47.6%) were admitted with complicated urinary tract infection, followed by intra-abdominal (19.5%) and respiratory tract infection (9.5%). Renal dysfunction was present in 71.5% of patients, while 28.6% had a moderate to severe Charlson Comorbidity Index score. Most patients (57.1%) had localized infections, whereas 23.8% developed sepsis. *Pseudomonas aeruginosa* (38.1%) was the most common pathogen, followed by *E. coli* and *Klebsiella spp*. (23.8% each). Urine (42.9%) and blood (28.6%) were the most frequent culture sites. Ineffective empirical antibiotics were received by 71.6% of patients, mainly for 4–8 days. Salvage therapy was initiated due to colistin resistance, contraindication, or intolerance, with patients receiving standard (57.1%) or renally adjusted (42.9%) CAZ-AVI + ATM dosing. Median treatment duration and hospital stay were 7 and 15 days, respectively. The 30-day follow-up revealed clinical stability in all patients, with one reinfection reported. Adverse events were minimal, including hypokalemia (9.5%) and mild eosinophilia (4.8%) (Table-I).

**Table-I:** Demographic and Clinical characteristics of patients with Carbapenem-resistant gram-negative infection (N=21)

Patient Demographics and Clinical Characteristics		
Age	Mean (SD)	37(21.9)
	Min-Max	1-85
Gender	Male	14(66.7)
	Female	7(33.3)
BMI	Mean (SD)	20.9(5.5)
	Min-Max	12.6-33.3
Monthly Income	Median (IQR)	60000(210000)
	Min-Max	25000-760000
Admitting Specialty	Infectious Disease	6(28.6)
	Urology	5(23.8)
	Pediatric	4(19)
	Nephrology	3(14.3)
	General Surgery	1(4.8)
	Internal Medicine	1(4.8)
	Orthopedics	1(4.8)
Admitting Diagnosis	Line Related Bacteremia	4(19)
	Complicated Intra-abdominal infection	3(14.3)
	Complicated UTI	10(47.6)
	Hospital Acquired Pneumonia (HAP)	2(9.5)
	Prosthetic joint infection	1(4.8)
	Soft Tissue Infection	1(4.8)
Comorbidities	Renal (CKD, ESRD)	10(71.4)
	On immune suppressive	3(21.4)
	Diabetes	2(14.3)
	Malignancy	2(14.3)
	Liver (CLD)	1(7.1)
Charlson Comorbidity Index	No Comorbid	12(57.1)
	Mild (1-2)	3(14.3)
	Moderate (3-4)	3(14.3)
	Severe (Greater than 5)	3(14.3)
Frailty Score	Median (IQR)	1(2)
	Min-Max	1-6
Severity	Localized infection	12(57.1)
	Systemic infection of moderate severity	7(33.3)
	Severe or life-threatening infection	2(9.5)
Organism	Pseudomonas aeruginosa	8(38.1)
	Escherichia coli	5(23.8)
	Klebsiella species	5(23.8)
	Burkholderia cepacia	1(4.8)
	Providenica	1(4.8)
	Serratia	1(4.8)
Culture Site	Urine	9(42.9)
	Blood	6(28.6)
	Respiratory	3(14.3)
	Tissue	3(14.3)
	Pus	3(14.3)
Empirical Therapy Prescribed	No Previous Medication	6(28.6)
	Inj, Colistimethate Sodium	4(19)
	Inj, Meropenem, Inj, Colistimethate Sodium	3(14.3)
	Inj, Cefaperazone + Sulbactam	2(9.5)
	Inj, Cefaperazone + Sulbactam, Inj, Colistimethate Sodium	2(9.5)
	Inj Ceftriaxone	1(4.8)
	Inj, Meropenem	1(4.8)
	Inj, Piperacillin + Tazobactam	1(4.8)
	Inj, Piperacillin + Tazobactam + Cap, Doxycycline	1(4.8)
Empirical Therapy Duration (N=14)	Less Than 3 Days	4(28.6)
	4 To 8 Days	9(64.3)
	Greater Than 9 Days	1(7.1)
Reason For Combination Therapy	Colistin Resistant	10(47.6)
	Colistin Contraindicated	9(42.9)
	Colistin Intolerant	2(9.5)
Dose Prescribed	Standard Dose	12(57.1)
	Renally Adjusted Dose	9(42.9)
Cost of Therapy for CAZ+AZT	Median (IQR)	256600 (136250-490200)
	Min-Max	29100-1518400
Duration of Treatment for CAZ+AZT	Median (IQR)	7(7)
	Min-Max	1-28
Length of Stay	Median (IQR)	15(13)
	Min-Max	1-74
Adverse Events	Hypokalemia	2(9.5)
	Eosinophilia	1(4.8)
	None	18(85.7)

### Clinical Outcomes of Study Participants:

Drug sensitivity profile showed that these bacterial isolates exhibited 57.1% resistance to Tigecycline, 76.1% to aztreonam, and 100% to CAZ-AVI. Furthermore, 47.6% had Colistin MIC in the intermediate or resistant range (Table-II)

**Table-II:** Drug sensitivity profile of the isolate

Drugs	Sensitive	Resistant
Carbapenem	-	21(100)
Tigecycline	9(42.9)	12(57.1)
Fosfomycin	4(19)	17(81)
Ceftazidime/Avibactam	-	21(100)
Aztreonam	5(23.8)	16(76.2)
Ceftriaxone	-	21(100)
Ciprofloxacin	-	21(100)
Amikacin	-	21(100)
Pipercillin-Tazobactum	-	21(100)
***Colistin MIC Value***		
Sensitive (<2)	8(38.1)	
Intermediate (2-4)	6(28.6)	
Resistant (>4)	4(19)	
Not Available	3(14.3)	

Out of 21 patients treated with CAZ-AVI and Aztreonam, three expired, where two had sepsis and were on Colistin-based therapy for over five days before starting this salvage treatment. Among the 18 survivors, only three patients showed no clinical response to the CAZ-AVI + Aztreonam therapy (Table-III).

**Table-III:** Clinical features of patients failing Salvage therapy

Age	Gender	Location of infection	Organism	Sepsis	Ventilation	Inotropes	Empirical therapy with duration	Reason to start CAZ+ATM	Duration of CAZ+ ATM (Days)	Outcome
40	Male	Bone&Joint	P.aeruginosa	Yes	No	Yes	Meropenem with colistin for 7 days	Colistin Contraindicated	11	Expired
46	Female	Abdominal	P.aeruginosa	No	No	No	Colistin for 5 days	Colistin Resistant	6	Expired
5	Female	Primary Line Related Bacteremia	Klebsiella species	Yes	Yes	Yes	Meropenem with colistin for 6 days	Colistin Resistant	6	Expired
29	Female	Urinary	Klebsiella species	No	No	No	Cefoperazone with Colistin for 5 days	Colistin Contraindicated	6	Not improved
25	Male	Disseminated	P.aeruginosa	No	No	No	Colistin	Colistin Intolerance	28	Not improved
41	Male	Abdominal	P.aeruginosa	No	No	No	None	Colistin Intolerance	7	Not improved

**Fig.1:**
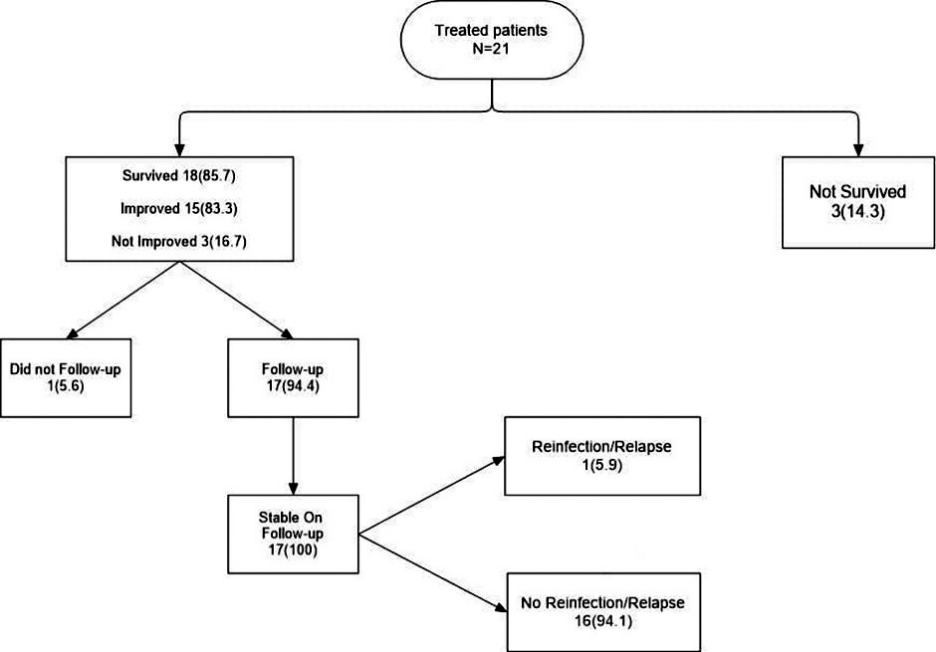
Clinical Outcome of salvage therapy.

Overall, Treatment outcomes of salvage therapy were favorable with an 85.7% survival rate and 83% of survivors showing clinical improvement (Fig.1).

## DISCUSSION

Rapid spread of Carbapenem resistance characterized by high mortality rates, extended hospitalizations, and staggering healthcare costs has become a major public health crisis in Pakistan. The latest antibiogram from the IHHN highlights alarming resistance rates to meropenem with 58% of *Klebsiella spp*., 41% of *Pseudomonas*, and 29% of *E. coli* isolates reported as resistant, underscoring severe scarcity of effective treatment options for multidrug-resistant Gram-negative infections in the country.

Amongst novel antibiotics available globally, Ceftazidime-Avibactam (CAZ-AVI) is the only currently accessible drug in Pakistan. However, increasing resistance and its lack of in-vitro activity against metallo-β-lactamase (MBL) producing organisms limit its utility.^10^,^12^ Similar demonstration of 100% resistance to CAZ-AVI was also found in the isolates under study, suggesting a predominance of MBL-producing strains (Table-II). Unfortunately, confirmation through carbapenemase detection was not possible due to unavailability of such testing at IHHN laboratory; a challenge commonly faced by laboratories across Pakistan. Absence of this diagnostic capability significantly hampers informed antimicrobial decision-making.

Colistin, historically considered a last-line agent, is also becoming less viable. Nearly half of the isolates in our study (47.6%) demonstrated high colistin MICs, consistent with national data reporting of rising colistin resistance. Additionally, 71.4% of patients in our cohort were unsuitable candidates for colistin therapy due to underlying renal impairment.^9^ This is particularly concerning given that colistin-associated nephrotoxicity has been reported in 26.7% to 34.8% of critically ill patients, further limiting its clinical usefulness.^13^,^14^

In response to these diagnostic and therapeutic challenges, a salvage therapy approach using a combination of Ceftazidime-Avibactam (CAZ-AV) and Aztreonam (ATM) was used to treat patients admitted with carbapenem-resistant Gram-negative infections. Notably, 71.6% of these patients had failed on prior empirical antibiotics due to antimicrobial resistance. Existing evidence suggests that combining CAZ-AVI with ATM is effective against approximately 90% of MBL producing *Enterobacterales* isolates.^15^,^16^ Although randomized clinical trial data are lacking, over 70 case reports support the clinical effectiveness of CAZ-AVI + ATM combination against CRE infections.^17^ Limited in-vitro studies and case reports indicate potential clinical efficacy against MBL-producing *Pseudomonas* infections.^18^ Importantly, current data shows comparable effectiveness between CAZ/AVI-ATM and ATM/AVI, making the former a critical option in regions such as South Asia where MBL-producing organisms are prevalent and therapeutic options are limited.^17^

The CAZ-AVI and ATM combination has been reported as safe used in both adult and pediatric populations.^19^ In our study, 19% of patients were children under five years of age Adverse effects were minimal, despite reports of transient transaminitis in early-phase studies.^20^ Furthermore, this combination has been associated with a markedly lower incidence of acute kidney injury (approximately 1.9%) compared to other treatment options, a key advantage in patients with pre-existing renal dysfunction.^21^

Our study demonstrated an overall clinical success rate of 71.5%. Treatment failure was observed in six patients, including three deaths and three survivors without clinical improvement. One patient developed reinfection one-month post-discharge, likely related to underlying renal stones and recent lithotripsy. Amongst non-survivors, two had severe sepsis and received inappropriate antimicrobial therapy for more than five days prior to initiation of salvage therapy, reflecting a high baseline risk of mortality. These outcomes compare favorably with published case series reporting success rates of approximately 60% at 30 days in carbapenem-resistant *Klebsiella* infections. 22

Evidence is surfacing that antibiotics targeting multiple penicillin-binding proteins (PBPs) may have enhanced activity. CAZ/AVI and ATM both target PBP3 primarily, but also bind to other PBPs (PBP1a, PBP1b, and PBP2), which may allow the combination to remain effective against MBL-producing *Enterobacterales* with altered PBP3, and together inhibit a broad range of β-lactamases.^17^ A study of CRE bacteremia showing 100% resistance to CAZ-AVI and high resistance to ATM found that CAZ-AVI +ATM combination treatment was associated with a significantly lower 30 days mortality rate in comparison to alternative regimens.^21^ A similar trend was also reflected in our findings where clinical success was achieved despite high resistance observed for both agents individually.

Treatment failures were predominantly observed in infections caused by *Pseudomonas aeruginosa*, consistent with emerging evidence showing limited activity of CAZ-AVI+ATM against this organism. 23 Recent IDSA guidelines recommend cefiderocol for MBL-producing *Pseudomonas* infections; however, its unavailability in Pakistan renders such infections effectively untreatable.^8^

Finally, the economic burden of therapy remains substantial. Globally, it has been reported that in-patient treatment cost of a single CRE infection can range from $22,484 to $66,031, with potential annual costs reaching $1.4 billion at higher incidence rates.^24^ The median treatment cost for CAZ-AVI+ATM in our study was PKR 256,600, far exceeding the median monthly income of PKR 60,000 among patients. Access to this therapy was possible only because IHHN provides care free of cost. This highlights an urgent need for regulatory support and funding mechanisms to ensure equitable access to life-saving antimicrobials in low- and middle-income countries.

### Strengths

This study being the first from Karachi, Pakistan, to evaluate salvage therapy, providing real-world evidence from a tertiary care setting, offers practical insights into the feasibility and clinical outcomes of this combination regimen in patients with multidrug-resistant infections with limited treatment options. Preliminary evidence provides first hand support for larger multicenter studies and may inform future clinical practice and regulatory decisions regarding use and availability of this therapy in low-resource settings.

### Limitations

As a pilot study there’s potential for bias due to its limited sample size. Absence of a comparative arm restricted direct evaluation against existing treatments while synergy testing between aztreonam and ceftazidime-avibactam could not be performed due to unavailability. These limitations underscore the need for larger, prospective studies to validate the findings.

## CONCLUSION

The study emphasizes potential of using CAZ-AVI + ATM combination in combating Gram-negative organisms that exhibit high-level resistance to nearly all major antibiotic classes. The favorable treatment outcomes observed warrant further investigation in comparative and prospective studies.

### Author Contribution:

**SS:** Conceptualized the study, developed study questionnaire, designed the methodology, Prepared and reviewed original draft. SS also supervised this study.

**SMZ:** Methodology design, study questionnaire design, literature search and writing the original draft of manuscript.

**NAS:** Data cleaning, methodology design, formal analysis, data visualization, and results interpretation.

**MHH and ZH** both contributed in data collection, literature search and manuscript writing.

All authors have read the final version and are responsible and accountable for the accuracy and integrity of the work.
